# The physical health of British adults with intellectual disability: cross sectional study

**DOI:** 10.1186/s12939-016-0296-x

**Published:** 2016-01-20

**Authors:** Eric Emerson, Chris Hatton, Susannah Baines, Janet Robertson

**Affiliations:** Centre for Disability Research, Lancaster University, Lancaster, UK; Centre for Disability Research and Policy, University of Sydney, Sydney, New South Wales Australia

**Keywords:** Health, Intellectual disability

## Abstract

**Background:**

Adults with intellectual disability have poorer health than their non-disabled peers. However, little is known about the health of the ‘hidden majority’ of adults with primarily mild intellectual disability who do not use intellectual disability services. The aims of the present study were: to estimate the physical health status of a population-based sample of British adults with and without mild intellectual disability while controlling for any potentially confounding effects resulting from between-group differences in gender, age, socio-economic disadvantage and neighborhood social capital.

**Methods:**

Secondary analysis of data from *Understanding Society*, a new longitudinal study focusing on the life experiences of UK citizens. We identified 299 participants aged 16–49 (1.2 % of the unweighted age-restricted sample) as having intellectual disability, and 22,927 as not having intellectual disability. Multivariate logistic regression was used to investigate between group differences adjusting for potential confounding personal characteristics (e.g., gender).

**Results:**

Unadjusted comparisons indicated that British adults with intellectual disability have markedly poorer health than their non-disabled peers on the majority of indicators investigated including self-rated health, multiple morbidity, arthritis, cancer, diabetes, obesity, measured grip strength, measured lung function and polypharmacy. Adjusting for between-group differences in age and gender had a marginal impact on these estimates. Further adjusting for between-group differences in socio-economic disadvantage and neighborhood quality had a more marked impact on estimates with the number of statistically significant differences reducing from 13 to 8 and statistically significant attenuation of odds on three indicators (self-rated health, SF-12 physical component and multiple morbidity).

**Conclusions:**

The ‘hidden majority’ of adults with primarily mild intellectual disability who do not use intellectual disability services have significantly poorer health than their non-disabled peers. This may, in part, reflect their increased risk of exposure to well established ‘social determinants’ of poorer health.

## Background

Intellectual disability refers to a significant general impairment in intellectual functioning that is acquired during childhood, typically operationalised as scoring more than two standard deviations below the population mean on a test of general intelligence [1]. While estimates of the prevalence of intellectual disability vary widely, it has been estimated that approximately 2 % of the adult population have intellectual disability [2, 3], the vast majority of who have mild intellectual disability (operationalised as an IQ in the range 50–69) [4]. The available evidence indicates that people with intellectual disability have significantly higher age adjusted rates of mortality and morbidity than their non-disabled peers [5–8]. This evidence, when combined with exposés of failings in healthcare systems [9–13] and increased attention to the human rights of disabled people [14], has led regulatory bodies and governments to stress the importance of reducing the health inequalities experienced by people with intellectual disability [12, 13, 15–17].

The evidence-base in this area is, however, significantly limited on two counts. First, by its reliance on convenience samples of adults with intellectual disabilities drawn from the population of people who use specialised health or welfare services for people with intellectual disabilities [5, 6]. This is problematic as the majority of adults with intellectual disability have mild intellectual disability and in most, if not all jurisdictions, do not use and are not known to intellectual disability services [18]. As a result, very little is currently known about the health or well-being of the group that has been termed the ‘hidden majority’ of adults with predominantly mild intellectual disability [19]. The available evidence does suggest, however, that adults with mild intellectual disability have significantly poorer health than their non-disabled peers and are also significantly more likely to be exposed to well-established social determinants of poor health [19–22]. Second, by the failure of most studies to take account of the extent to which any differences in health status may be attributable to potential confounding variables such as differential rates of exposure to common social determinants of poorer health including socio-economic disadvantage and low neighborhood social capital rather than intellectual disability per se [6].

The aims of the present study were: (1) to estimate the physical health status of a population-based sample of British adults with and without mild intellectual disability; while (2) controlling for any potentially confounding effects resulting from between-group differences in gender, age, socio-economic disadvantage and neighborhood social capital.

## Methods

The present study involved secondary analysis of data collected in the first four Waves of *Understanding Society*, a new longitudinal study focusing on the life experiences of UK citizens. Data were downloaded from the UK Data Archive (http://www.data-archive.ac.uk/). Full details of the surveys’ development and methodology are available in a series of reports [23–30], key aspects of which are summarized below.

### Samples

In the first wave of data collection (undertaken between January 2009 and December 2011), random sampling from the Postcode Address File in Great Britain and the Land and Property Services Agency list of domestic properties in Northern Ireland identified 55,684 eligible households. Interviews were completed with 50,994 individuals aged 16 or older from 30,117 households, giving a household response rate of 54 % and an individual response rate within co-operating households of 86 % [23, 29]. Participants are followed up annually. Sample sizes for subsequent Waves are: Wave 2 (January 2010 and March 2012) 54,584 individuals from 30,428 households; Wave 3 (January 2011 and July 2013) 49,708 individuals from 27,715 households; and Wave 4 (January 2012 and June 2014) 47,132 individuals from 25,814 households [30]. Longitudinal individual re-interview rates have risen consistently from 75 % (between Waves 1 and 2) to 85 % (between Waves 3 and 4) [30].

### Procedures

Data collection for variables used in the present paper was undertaken using either Computer Assisted Personal Interviewing or by examination conducted by a visiting nurse (see below).

### Measures

#### Intellectual disability

*Understanding Society* does not include information on the formal diagnosis of intellectual disability. As a result, we identified adults with intellectual disability on the basis of the results of cognitive testing undertaken at Wave 3 and self-reported educational attainment. The vast majority of children with intellectual disability have very low educational attainment [31]. As a result, low self-reported educational attainment (no educational qualifications) was used as a selection criterion as evidence that low cognitive ability may have originated in childhood (one of the defining characteristics of intellectual disability). Due to historical changes in educational qualifications and attainment in the UK, we restricted our analysis to the age range 16–49.

In Wave 3 a battery of five cognitive tests was used to assess memory (two tests) and cognitive functioning (three tests; Number Series, Verbal Fluency, Numerical Ability) [32]. The Number Series test was developed for use in the US Health and Retirement Study (HRS) [33]. The Verbal Fluency test has been used in the English Longitudinal Study of Ageing (ELSA) [34], the German Socio-economic Panel Study [35] and the National Survey of Health and Development [36]. The Numerical Ability test was taken from ELSA and some portions of it have been used in the HRS and Survey of Health, Ageing and Retirement in Europe [37].

First, we standardized test scores on the latter three tests to have a mean of zero and standard deviation of one. Second, we used linear regression to impute missing standardized test scores from obtained scores on completed tests. No other variables were used in the imputation process. This led to the imputation of Numeric Ability scores for 153 participants (0.7 % of the used sample), Verbal Fluency scores for 141 participants (0.6 %) and Number Series scores for 1,214 participants (4.9 %). Third, we used principal components analysis to extract the first component (which accounted for 63 % of the variance) from the three scales as an estimate of general intelligence [38]. Fourth, we identified participants as having intellectual disability if they scored lower than two standard deviations below the mean on the extracted component (the conventional cut-off point for defining intellectual disability used in ICD-10) and had no educational qualifications. This identified 294 participants (1.2 % of the unweighted age-restricted sample) as having intellectual disability. An additional 532 participants scored less than two standard deviations below the mean on the extracted component but did have educational qualifications.

Fifth, we included in the intellectual disability group five participants who gave consent for testing but for whom all three tests were terminated due to their inability to understand the test instructions, and also had no educational qualifications. The complete procedure identified 299 participants (1.2 % of the unweighted age-restricted sample) as having intellectual disability.

### Health: self-report

Four forms of self-reported health data were collected. First, the SF-12 was used to assess physical and mental health [39]. We derived a binary measure of SF-12 Physical Health on the basis of Wave 3 responses to the SF12 Physical Component scores falling within the bottom decile of the weighted Wave 3 sample. Second, self-rated health was evaluated by a single question incorporating five possible response options: ‘*In general, would you say your health is … (1) excellent, (2) very good, (3) good, (4) fair, (5) poor*’. We converted these data into a binary measure of ‘poor’ vs. better than ‘poor’ health.

Third, in Waves 1–4 participants were asked ‘*Has a doctor or other health professional ever told you that you have any of the conditions listed on this card?*’ Response options included: asthma, arthritis, congestive heart failure, coronary heart disease, angina, heart attack or myocardial infarction, stroke, emphysema, hyperthyroidism or an over-active thyroid, hypothyroidism or an under-active thyroid, chronic bronchitis, any kind of liver condition, cancer or malignancy, diabetes, epilepsy, high blood pressure. We combined data across Waves 1–4 to derive lifetime prevalence rates of each health condition. Due to very low prevalence rates of specific conditions we derived a measure of respiratory disorder (one or more of emphysema or chronic bronchitis), other cardio-vascular disease (one or more of congestive heart failure, coronary heart disease, angina, heart attack or myocardial infarction, stroke) and thyroid condition (one or more of hyperthyroidism or an over-active thyroid, hypothyroidism or an under-active thyroid).

Finally, participants were asked if since the previous Wave they had had a hospital admission for any newly diagnosed health conditions (using the list of conditions presented above). We combined data across Waves 2 to 4 to derive a variable of hospitalization for a newly diagnosed condition.

### Health: biosocial data collected by trained nurse

In Waves 2 and 3 of Understanding Society biosocial data were collected by a trained nurse from a subsample of 11,142 respondents [40], including 60 respondents (0.5 %) who we had identified as having intellectual disability. In Wave 2, the nurse health assessments were conducted with a subset of the General Population Sample component of Understanding Society, with data collection extending over 24 months. In Wave 3, the measures were undertaken with the British Household Panel Survey sample component of Understanding Society. The eligibility criteria in both Waves were completion of a full face-to-face interview in the corresponding wave, being aged 16 or older, living in England, Scotland or Wales, and completion of the interview in English. In the second year of Wave 2, eligibility was further restricted to .81 of the primary sampling units in England. Nurse visits were not conducted in Northern Ireland or with members of the ethnic minority boost sample. Women who were pregnant at the time of the nurse assessment were classified as not eligible.

The measures taken included: (1) measured weight and height from which BMI was calculated and obesity categorized as a BMI of 30 or more; (2) systolic and diastolic blood pressure; (3) dominant hand grip strength; (4) lung function; (5) medications taken. A measure of stage 2 hypertension or on medication was derived from systolic and diastolic blood pressure readings of greater than 160/100 or that the participant was taking medication for hypertension. Four measures of lung function were derived from the available data: (1) forced expiratory volume in one second (FEV1); (2) forced vital capacity (FVC), the total amount of air (in liters) that can forcibly be blown out after a full inspiration; (3) the ratio of FEV1 to FVC (FEV1/FVC ratio); and (4) peak expiratory flow (PEF), the speed of air moving out of the lungs at the beginning of expiration measured in litres per second. Polypharmacy was defined from the medications data as taking five or more separate medications [41].

### Socio-economic disadvantage

We used two indicators of socio-economic disadvantage. Self-assessed financial status was assessed at Wave 3 by a single item: ‘How well would you say you yourself are managing financially these days? Would you say you are… 1 Living comfortably, 2 Doing alright, 3 Just about getting by, 4 Finding it quite difficult or 5 finding it very difficult?’ Material hardship was assessed at Wave 1 by summing ‘cannot afford’ (questions one to seven) or ‘no’ responses (question eight) to eight questions preceded by the introduction ‘*Next we have some questions about the sorts of things that some families/people have but which many people have difficulty finding the money for. For each of the following things please tell me the number from the showcard which best explains whether you (and your family/partner) have it or not. Do you (and your family partner) have: (1) Friends or family around for a drink or meal at least once a month? (2) Two pairs of all weather shoes for all adult members of the family? (3) Enough money to keep your house in a decent state of repair? (4) Enough money to make regular savings of £10 a month or more for rainy days or retirement? (5) Household contents insurance? (6) Enough money to replace any worn out furniture? (7) Enough money to replace or repair major electrical goods such as a refrigerator or a washing machine, when broken? (8) For the next question please just answer yes or no. In winter, are you able to keep this accommodation warm enough?*’

### Neighborhood social capital

A scale of neighborhood social capital was derived from 13 items relating to perceptions of neighborhood quality and civic and social participation (in the following list #3 contains four separate items):

1. ‘*Overall, do you like living in this neighborhood (Yes/No)?*’
2. ‘*Are you able to access all services such as healthcare, food shops or learning facilities when you need to (Yes/No)?*’
3. ‘*I am going to read out a set of statements that could be true about your neighborhood. Please tell me how much you agree or disagree that each statement describes your neighborhood (1 Strongly agree, 2 Agree, 3 Neither agree nor disagree, 4 Disagree, 5 Strongly disagree): (a) First, this is a close-knit neighborhood; (b) People around here are willing to help their neighbors; (c) People in this neighborhood can be trusted; (d) People in this neighborhood generally don't get along with each other.*’ Data were recoded into binary variables; 1–2 v 3–5 for positively worded questions (a-c), 1–3 v 4–5 for question (d).
4. ‘*Now I have some questions about crime. Do you ever worry about the possibility that you, or anyone else who lives with you, might be the victim of crime? Is this a big worry, a bit of a worry, or an occasional doubt?*’ Data were recoded into a binary variable; crime is a big worry v not.
5. ‘*How safe do you feel walking alone in this area after dark? (1 Very safe, 2 Fairly safe, 3 A bit unsafe, 4 Very unsafe, 5 SPONTANEOUS: Never goes out after dark)*’. Data were recoded into a binary variable fairly safe/very safe v not.
6. ‘*How many close friends would you say you have?*’ Data were recoded into a binary variable; two or more close friends v not.
7. ‘*Do you go out socially or visit friends when you feel like it (Yes/No)?*’
8. ‘*Please tell me how easy or difficult you would find it to visit family or relatives when you need to (1 Very difficult, 2 Difficult, 3 Neither difficult nor easy, 4 Easy, 5 Very easy, 6 Has no family).*’ Data were recoded into a binary variable; Easy/very easy v not.
9. ‘*Are you currently a member of any of the kinds of organizations on this card (1 Political party, 2 Trade Unions, 3 Environmental group, 4 Parents'/School Association, 5 Tenants'/Residents' Group or Neighborhood Watch, 6 Religious group or church organization, 7 Voluntary services group, 8 Pensioners group/organization, 9 Scouts/Guides organization, 10 Professional organization, 11 Other community or civic group, 12 Social Club/Working men's club, 13 Sports Club, 14 Women's Institute/Townswomen's Guild, 15 Women's Group/Feminist Organization, 16 Other group or organization*)’. Data were recoded into a binary variable; member of one or more organization vs. not.
10. Whether the informant was employed for 16 hours a week or longer

Exploratory analysis of the resulting data indicated that the recoded binary variables showed acceptable internal consistency (alpha = 0.61). As a result, they were combined into a scale of ‘neighborhood social capital’ (range 0–13).

### Approach to analysis

Our approach to analysis was undertaken in four stages. First, we recoded all ordinal or scale outcome variables to binary variables. The reasons underlying this decision were: (1) to allow for a common metric of strength of association (odds ratios) to be used across all outcome variables; (2) to avoid analytical problems associated with the non-normal distribution of many of the outcome variables [42]. To create the binary outcome variables we defined low functioning on a particular variable as falling within the bottom 10 % of the population weighted sample. This decision was based on the absence of well-established clinical cut-offs for individual scales and tests. However, functioning in the lowest population decile is likely to be of clinical concern. For example, current UK guidelines suggests that a diagnosis of COPD is indicated when the FEV_1_/FVC ratio is less than 0.7 and the FEV_1_ is less than 80 % of predicted [43]. In the present study, the cut-off point for the lowest sample decile for the FEV_1_/FVC ratio was 0.7.

Second, we made simple bivariate comparisons between participants with and without intellectual disability with regard to available socio-demographic characteristics that may have a potential association with health (e.g., financial strain, gender). Again, to allow for a common metric of strength of association to be used across demographic variables we converted all ordinal or scale variables to binary variables using the sample median as the cut-off (age 16–33, 34–49; self-assessed financial status ‘living comfortably’ or ‘doing alright’/not; material hardship 0,1+; neighborhood social capital (low 0–9, 10–13).

Third we made unadjusted bivariate comparisons (using binary logistic regression) between participants with and without intellectual disability with regard to health status.

Fourth, we made adjusted bivariate comparisons (using multivariate binary logistic regression) between participants with and without intellectual disability with regard to health status adjusted to take account in between-group differences in potential confounding variables. In Model 1 we controlled for between sample differences in age, gender and (for self-report measures) the number of waves in which the respondent participated. In Model 2 we controlled for between sample differences in exposure to two well-established social determinants of poorer health; socio-economic disadvantage [44, 45] and neighborhood social capital [46–48].

We report effect size categories for Odds Ratios following the recommendations of Olivier and Bell; small (OR < =0.82 or > =1.22), medium (OR < =0.54 or > =1.86), large (OR < =0.33 or > =3.00) [49]. All analyses were undertaken using SPSS 20.

### Ethical approval

Understanding Society is designed and conducted in accordance with the ESRC Research Ethics Framework and the ISER Code of Ethics. The University of Essex Ethics Committee approved Waves 1–5 of Understanding Society. Approval from the National Research Ethics Service was obtained for the collection of biosocial data by trained nurses in Waves 2 and 3 of the main survey (Understanding Society – UK Household Longitudinal Study: A Biosocial Component, Oxfordshire A REC, Reference: 10/H0604/2).

## Results

In the first stage of analysis we made simple bivariate comparisons between participants with and without intellectual disability with regard to selected demographic characteristics that could have a potential association with health. In the full sample participants with intellectual disability were significantly more likely than other participants to be aged 34 or over (66 % v 53 %, OR = 1.73(1.36-2.21), *p* < 0.001), to have low self-assessed financial status (‘just getting by’ or ‘finding it difficult/very difficult’; 63 % v 43 %, OR = 2.33(1.82-2.94, *p* < 0.001), to be unable to afford at least one item (72 % v 53 %, OR = 2.22(1.72-2.86, *p* < 0.001) and to experience low neighborhood social capital (73 % v 45 %, OR = 3.33(2.56-4.35, *p* < 0.001). There was also a non-significant trend for them to be women (66 % v 53 %, OR = 1.26(0.99-1.59). Similar differences were observed in the nurse measurement subsample. The associations between intellectual disability and low neighborhood social capital were indicative of a large effect size and the associations between intellectual disability and socio-economic disadvantage were indicative of medium effect sizes.

As a result of these marked between-group differences, all subsequent estimates of effect sizes are adjusted to take account of between-group differences in age, gender, socio-economic disadvantage and neighborhood quality. The adjusted comparisons took account of potential confounding variables in two stages. In stage one we controlled for between sample differences in age, gender and (for self-report measures) the number of waves in which the respondent participated. In stage two we controlled for between sample differences in socio-economic disadvantage and neighborhood social capital. The results of these analyses are presented in Table 1.

**Table 1:** The physical health of adults with intellectual disability

	Prevalence (ID)	Prevalence (no ID)	Unadjusted Odds	Model 1: Odds adjusted for age and gender	Model 2: Odds adjusted for age, gender, socio-economic disadvantage and neighborhood social capital
General Physical Health (W3)					
‘Poor’ self-rated health	13 %	3 %	4.40*** (3.12-6.21)	3.89*** (2.74-5.53)	1.92*** (1.32-2.79)
SF-12 Physical Component (bottom 10 % of weighted sample)	28 %	10 %	3.66*** (2.61-5.12)	3.49*** (2.48-4.91)	2.20*** (1.54-3.14)
Self-Reported Health Conditions (W1-4)					
Asthma	9 %	12 %	0.80 (0.53-1.21)	0.85 (0.56-1.29)	0.75 (0.49-1.14)
Arthritis	7 %	3 %	2.86*** (1.77-4.61)	2.31** (1.42-3.76)	1.52 (0.92-2.51)
Cancer or malignancy	2 %	1 %	2.64* (1.07-6.49)	**2.14** (0.87-5.29)	1.65 (0.66-4.17)
Diabetes	6 %	2 %	4.53*** (2.77-7.40)	3.90*** (2.37-6.40)	2.44** (1.47-4.07)
Epilepsy	2 %	1 %	2.52* (1.11-5.74)	2.53* (1.11-5.76)	1.67 (0.72-3.84)
Thyroid disorder	2 %	2 %	1.20 (0.49-2.93)	0.95 (0.39-2.33)	0.78 (0.31-1.93)
Respiratory disorder^a^	1 %	1 %	1.74 (0.55-5.51)	1.44 (0.45-4.57)	0.78 (0.24-2.52)
High blood pressure	5 %	5 %	1.04 (0.60-1.79)	0.87 (0.50-1.51)	0.69 (0.40-1.20)
Other CVD^b^	3 %	1 %	3.91*** (1.90-8.02)	3.34** (1.62-6.89)	**2.02** (0.97-4.23)
Any morbidity	24 %	22 %	1.18 (0.90-1.55)	1.27 (0.96-1.67)	1.09 (0.82-1.44)
Multiple morbidity					
2+	8 %	4 %	2.63*** (1.72-4.00)	2.25*** (1.47-3.42)	1.39 (0.90-2.16)
3+	2 %	1 %	2.45* (1.08-5.57)	**2.00** (0.88-4.57)	1.02 (0.44-2.39)
Hospital admission for newly diagnosed condition (W2-4)	7 %	2 %	2.64*** (1.51-4.59)	2.53** (1.45-4.41)	1.98* (1.13-3.49)
Nurse measures (W2 or 3)					
Obese	42 %	26 %	2.12** (1.25-3.60)	2.12** (1.24-3.62)	1.67 (0.97-2.87)
Stage 2 hypertension or on medication	2 %	4 %	0.56 (0.08-4.10)	0.51 (0.07-3.75)	**0.32** (0.04-2.40)
Grip strength for dominant hand (bottom 10 % of weighted sample)	32 %	9 %	4.46*** (2.50-7.95)	7.10*** (3.64-13.98)	5.94*** (2.98-11.84)
Lung function					
Fev1 (bottom 10 % of weighted sample)	30 %	9 %	4.53*** (2.40-8.52)	5.67*** (2.78-11.56)	4.34*** (2.10-8.96)
Fvc (bottom 10 % of weighted sample)	28 %	8 %	4.56*** (2.38-8.68)	6.79*** (3.12-14.77)	5.13*** (2.33-11.29)
Fev1/fvc ratio (bottom 10 % of weighted sample)	17 %	10 %	**1.90** (0.88-4.08)	1.77 (0.81-3.84)	1.57 (0.72-3.45)
Peak expiratory flow (bottom 10 % of weighted sample)	37 %	8 %	6.76*** (3.69-12.36)	9.50*** (4.89-18.48)	7.14*** (3.63-14.04)
Polypharmacy (taking 5+ meds)	8 %	4 %	2.56* (1.02-6.44)	**2.40** (0.94-6.13)	1.15 (0.44-3.05)
Polypharmacy (taking 5+ meds)^c^				**2.07** (0.74-5.74)	1.19 (0.42-3.40)

Odds: Odds ratio with 95 % confidence intervals. Odds ratios in bold equivalent to moderate or large effect size

*p < 0.05, **p < 0.01, ***p < 0.001

^a^One or more of emphysema or chronic bronchitis

^b^One or more of congestive heart failure, coronary heart disease, angina, heart attack or myocardial infarction, stroke

^c^Also adjusting for number of health conditions

Inspection of the unadjusted estimates indicates that all 16 statistically significant differences and all 17 differences representing a moderate or larger effect size were associated with poorer health among participants with intellectual disability. Adjusting for between-group differences in age and gender generally had a marginal impact on odds estimates. However, the statistical significance of between-group differences in the unadjusted comparisons were eliminated for three outcomes (cancer or malignancy, multimorbidity, polypharmacy). All remaining statistically significant differences and differences representing a moderate or larger effect size were associated with poorer health among participants with intellectual disability.

Adjusting for between-group differences in socio-economic disadvantage and neighborhood quality had a more marked impact on odds estimates. The number of statistically significant differences reduced from 13 to 8 (statistical significance eliminated for arthritis, epilepsy, other cvd, multiple morbidity 2+, obesity) and the number of differences representing a moderate or larger effect size reduced from 16 to 10. On three indicators (self-rated health, SF-12 physical component and multiple morbidity) adjusting for between-group differences in socio-economic disadvantage and neighborhood quality significantly attenuated the relationship between intellectual disability and health (the point further adjusted estimate lay outside of the 95 % confidence intervals for the pre-adjusted estimate). On one indicator (stage 2 hypertension or on hypertensive medication) participants with intellectual disability were at lower odds (large effect size), although the difference was not statistically significant.

## Discussion

Our results indicate that in unadjusted comparisons British adults with intellectual disability have markedly poorer health than their non-disabled peers on the majority of indicators investigated including self-rated health, multiple morbidity, arthritis, cancer, diabetes, obesity, measured grip strength, measured lung function and polypharmacy. Adjusting for between-group differences in age and gender had a marginal impact on odds estimates. Further adjusting for between-group differences in socio-economic disadvantage and neighborhood quality had a more marked impact on odds estimates with the number of statistically significant differences reducing from 13 to 8 and statistically significant attenuation of odds on three indicators (self-rated health, SF-12 physical component and multiple morbidity).

These results add to existing knowledge about the health inequalities faced by people with intellectual disability in three important ways. First, they are based on the analysis of contemporary population-based sampling frames, a relative rarity in this field of study [6]. Second, the study included direct assessment of health status in such areas as obesity, hypertension, grip strength, lung function and polypharmacy, again a relative rarity in this field of study. Finally, being based on samples drawn from general households, participants are likely to include adults with less severe intellectual disability who may not be in receipt of specialized disability services. Most intellectual disability research is based on convenience samples drawn from the users of specialized disability services (typically people with more severe intellectual disability). As a result, very little is currently known about the health or well-being of the group that has been termed the ‘hidden majority’ of adults with predominately mild intellectual disability [19, 20, 50].

The results are broadly consistent with the existing literature in documenting the poorer health status of people with intellectual disabilities when compared to their non-disabled peers [5–8]. The one potentially anomalous result was that participants with intellectual disability were at lower odds of stage 2 hypertension or taking hypertensive medication than their peers. However, while the association was indicative of a large effect size, the difference was not statistically significant. Indeed, previous research has indicated that hypertension among people with intellectual disabilities is similar to or possibly higher than that of the general population [51–53].

However, there are seven limitations to the study that should be kept in mind when considering the salience and implications of these results. First, while intellectual disability was identified on the basis of tests of cognitive ability, we have only indirect evidence (through reported lack of educational attainment) that their cognitive impairments may have originated in childhood. Second, the use of a general household sampling frame excludes people with (primarily more severe) intellectual disability living in institutional forms of residential care. Third, the consent and interview procedures used in *Understanding Society* are also likely to exclude people with more severe intellectual disability from participating. Consequently, the results are likely to be particularly relevant to understand the health of British adults with less severe intellectual disability. Fourth, no reasonable adjustments were made to the interview process to take account of possible intellectual impairments among participants. As a result, some participants with intellectual disability may have found some questions confusing, reducing the validity of their responses. Fifth, participants with intellectual disability were markedly under-represented in the nurse measurement sample (0.5 %) when compared to the main interview sample (1.2 %). While the reasons for under-representation are unknown, the low level of participation of adults with intellectual disability in the nurse measurement sample: (1) produced a small sample size with associated risk of significantly underpowered analyses; and (2) may have introduced biases that were not controlled for in the sample weighting procedure. Sixth, it was not possible to include other potentially confounding variables in the model (e.g., exposure to discrimination [54, 55]).

Finally, while the cross-sectional analyses presented in this paper are consistent with the hypothesis that the poorer health of adults with mild intellectual disability may be partially attributable to their living conditions, the cross-sectional nature of the data do not allow us to rule out other explanations. For example, poor health may lead to downward social mobility [56], or factors associated with intellectual disability (e.g., low literacy) may lead independently to socio-economic disadvantage/social exclusion and poor health [6].

## Conclusions

As noted in the Rio Declaration [57] and the recent WHO report on the health divide in Europe [58], action on addressing the social determinants of health needs to pay particular attention to the situation of marginalized or vulnerable groups. This paper addresses the social determinants of health inequity of a particularly marginalized and vulnerable group (adults with intellectual disability). The results are consistent with the hypothesis that the poorer health of this group may (in part) be related to their poorer living conditions, rather than their intellectual impairments per se.
